# Supplementary material for: Stacking Model‐Based Classifiers for Dealing With Multiple Sets of Noisy Labels
Source: Biom J. 2025 Mar 12;67(2):e70042. doi: 10.1002/bimj.70042 (PMC11898607; doi:10.1002/bimj.70042)
Supplement: Supplementary file 1 — Supporting Information [file BIMJ-67-e70042-s001.zip › density-based-ensemble-model-main/competitors_real_data/netlab-master/netlab-master/help/linef.htm]

Netlab Reference Manual linef


# linef

## Purpose

Calculate function value along a line.

## Description

`linef(lambda, fn, x, d)` calculates the value of the function
`fn` at the point `x+lambda*d`. Here `x` is a row vector
and `lambda` is a scalar.

`linef(lambda, fn, x, d, p1, p2, ...)` allows additional
arguments to be passed to `fn()`.
This function is used for convenience in some of the optimisation routines.

## Examples

An example of
the use of this function can be found in the function `linemin`.

## See Also

`gradchek`, `linemin`

---

**Pages:**
Index


---

Copyright (c) Ian T Nabney (1996-9)
